# Beneficial effects of exercise on gut microbiota functionality and barrier integrity, and gut-liver crosstalk in an *in vivo* model of early obesity and non-alcoholic fatty liver disease

**DOI:** 10.1242/dmm.039206

**Published:** 2019-04-30

**Authors:** Sara Carbajo-Pescador, David Porras, María Victoria García-Mediavilla, Susana Martínez-Flórez, María Juarez-Fernández, María José Cuevas, José Luis Mauriz, Javier González-Gallego, Esther Nistal, Sonia Sánchez-Campos

**Affiliations:** 1 Instituto de Biomedicina (IBIOMED), Universidad de León, 24071, León, Spain; 2 Centro de Investigación Biomédica en Red de Enfermedades Hepáticas y Digestivas (CIBERehd), Instituto de Salud Carlos III, Spain; 3 Servicio de Aparato Digestivo del Complejo Asistencial Universitario de León, 24071, León, Spain

**Keywords:** Childhood obesity, Fecal metabolome, Gut-liver axis, Intestinal microbiota, Metabolic syndrome

## Abstract

Childhood obesity has reached epidemic levels, representing one of the most serious public health concerns associated with metabolic syndrome and non-alcoholic fatty liver disease (NAFLD). There is limited clinical experience concerning pediatric NAFLD patients, and thus the therapeutic options are scarce. The aim of this study was to evaluate the benefits of exercise on gut microbiota composition and functionality balance, and consequent effects on early obesity and NAFLD onset in an *in vivo* model. Juvenile (21-day-old) male Wistar rats fed a control diet or a high-fat diet (HFD) were subjected to a combined aerobic and resistance training protocol. Fecal microbiota was sequenced by an Illumina MiSeq system, and parameters related to metabolic syndrome, fecal metabolome, intestinal barrier integrity, bile acid metabolism and transport, and alteration of the gut-liver axis were measured. Exercise decreased HFD-induced body weight gain, metabolic syndrome and hepatic steatosis, as a result of its lipid metabolism modulatory capacity. Gut microbiota composition and functionality were substantially modified as a consequence of diet, age and exercise intervention. In addition, the training protocol increased *Parabacteroides*, *Bacteroides* and *Flavobacterium* genera, correlating with a beneficial metabolomic profile, whereas *Blautia*, *Dysgonomonas* and *Porphyromonas* showed an opposite pattern. Exercise effectively counteracted HFD-induced microbial imbalance, leading to intestinal barrier preservation, which, in turn, prevented deregulation of the gut-liver axis and improved bile acid homeostasis, determining the clinical outcomes of NAFLD. In conclusion, we provide scientific evidence highlighting the benefits of gut microbiota composition and functionality modulation by physical exercise protocols in the management of early obesity and NAFLD development.

## Abstract (precis)

**Summary:** The beneficial effects of exercise against diet-induced early obesity and NAFLD are mediated by its capacity to modulate intestinal microbiota composition and functionality, restore lipid metabolism and prevent disruption of the gut-liver axis.

## INTRODUCTION

Childhood obesity has emerged as one of the most serious public health issues from this century, largely as a result of unhealthy dietary habits and sedentary patterns in children. Moreover, it is known that overweight children and adolescents tend to remain obese in adulthood and have greater susceptibility to developing metabolic disorders during their lifetime (Abarca-Gómez et al., 2017).

In line with the increase in obesity in children, non-alcoholic fatty liver disease (NAFLD) represents the main cause of chronic hepatic disorders, with an alarming prevalence of 40-70% among overweight/obese children living in Western countries (Panera et al., 2018). In adults, NAFLD condition is considered the hepatic manifestation of the metabolic syndrome, associated with insulin resistance, fat infiltration in >5% of hepatocytes (steatosis), deregulated lipid metabolism, oxidative stress response and subsequent hepatotoxicity (Tiniakos et al., 2010). These hallmarks, together with gut microbiota and dysfunction of the gut-liver axis, are critical in the progression through more severe inflammatory and fibrotic stages of steatohepatitis (Tiniakos et al., 2010). NAFLD development during periods of active growth and puberty is not exactly identical to that in adults. Furthermore, there is less clinical experience with pediatric NAFLD patients and, because no perfect NAFLD *in vivo* model has been developed, the pathogenesis of pediatric NAFLD remains unclear. Consequently, therapeutic options are scarce with respect to safety, effectiveness and patient compliance (Giorgio et al., 2013).

Growing scientific evidence supports the importance of gut microbiota in metabolic disorders linked to obesity and liver diseases (Bashiardes et al., 2016; Ma et al., 2017). Gut microbiota is known to improve the host energy yield from digested food, to alter choline metabolism and to regulate the enterohepatic circulation of bile acids (BAs) (Porras et al., 2018). Likewise, changes in the microbiota composition are known to disrupt the intestinal barrier integrity, allowing the subsequent translocation of bacterial products into the portal vein to alter the gut-liver crosstalk, which induces downstream inflammatory pathways involved in NAFLD progression (Brandl and Schnabl, 2015). The complex relationship between gut microbiota and NAFLD opens up an attractive window for seeking successful and safe NAFLD therapies.

Healthy dietary habits and physical activity are prescribed to obese patients, resulting in improvements in insulin sensitivity, lipid metabolism, hepatic function and liver steatosis (Ordoñez et al., 2015). Recent studies have associated the beneficial metabolic effects of exercise with its capacity to reshape microbiota composition in animal models and adults (Cerdá et al., 2016; Codella et al., 2018; Evans et al., 2014), suggesting that exercise practice in NAFLD patients might be a potential strategy to modulate intestinal bacterial composition and improve metabolic status. However, no studies in pediatric patients have been conducted to date. Consequently, we performed this novel study with high-fat diet (HFD)-fed juvenile rats as an *in vivo* model to evaluate the effects of exercise performance on early obesity and NAFLD onset. We aimed to improve the understanding of the complex relationship between physical activity, obesity-related disorders, and gut microbiota composition and functionality during early growth. Results from this research will provide scientific evidence supporting the use of physical exercise protocols in the management of childhood obesity and NAFLD development.

## RESULTS

### Exercise attenuates HFD-induced metabolic syndrome and liver injury in our *in vivo* model of early obesity and NAFLD

To assess the effects of exercise training on HFD-induced metabolic syndrome and obesity-related childhood NAFLD, 21-day-old Wistar rats were fed a control diet or HFD for 6 weeks and subsequently split into four subgroups, so that half of the rats from each diet group remained sedentary and the other half undertook a training protocol for an additional 5 weeks (see Materials and Methods). After the first 6 weeks into the HFD-based regimen, pubertal male rats demonstrated an increase in body weight and insulin resistance [determined by the homeostatic model assessment of insulin resistance (HOMA-IR)] (Fig. S1A,B), markers of early obesity and metabolic syndrome as described for pediatric patients (Giorgio et al., 2013).

Citrate synthase activity was assessed as a proof of concept to verify exercise effectiveness. The combined protocol of aerobic and resistance training positively increased the citrate synthase enzymatic activity, suggesting an improvement in the oxidative metabolism capacity after 5 weeks of exercise performance. No remarkable differences in citrate synthase activity were found between control and HFD-fed sedentary rats (Fig. S1C).

**Fig. 1.**
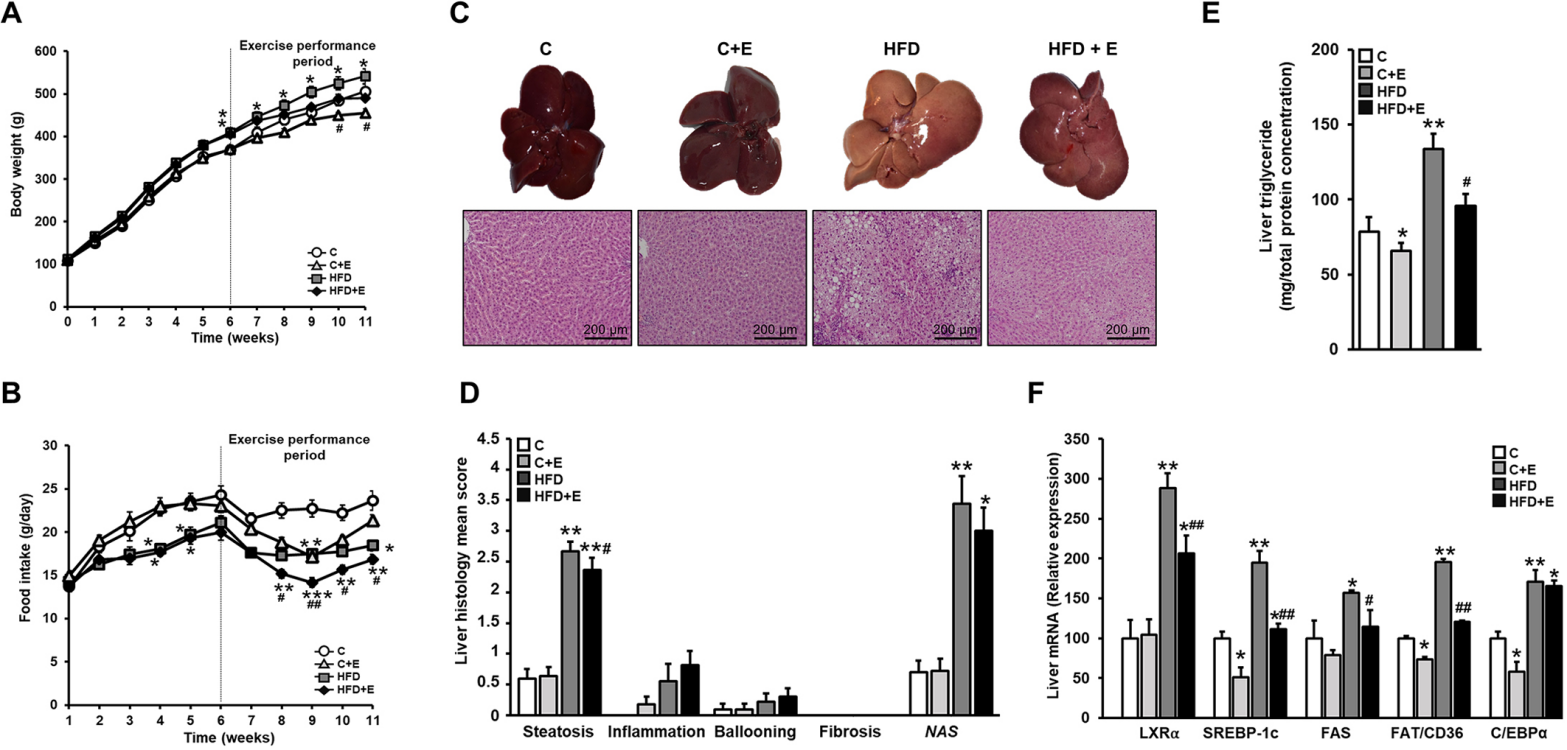
**Effects of diet and exercise intervention on obesity, NAFLD-associated hepatic histological findings and lipid-metabolism regulation in juvenile rats fed a control diet or HFD over 11 weeks.** (A) Body weight. (B) Food intake. (C) Top: liver specimens. Bottom: representative H&E-stained liver sections. Scale bar: 200 μm. (D) NAFLD activity score (NAS) (calculated from individual scores for steatosis, lobular inflammation and ballooning). (E) Intrahepatic triglyceride content. (F) mRNA levels of hepatic lipid-metabolism-related genes, determined by quantitative real-time PCR (RT-qPCR) at the end of the study (week** **11). Data are means±s.e.m. (*n*=12 rats per group). *P<0.05, **P<0.01, ***P<0.001 versus control; ^#^*P*<0.05, ^##^*P*<0.01 versus HFD. C, control diet; C+E, control diet and exercise training; HFD, high-fat diet; HFD+E, high-fat diet and exercise training.

The effects of diet and exercise on body weight gain, food intake, metabolic syndrome, and liver function and damage-related parameters at the end of the study are shown in Fig. 1A,B and Table 1. Significant differences between the weights of control and HFD-fed rats appeared at week 6 (beginning of the training protocol) (Fig. 1A). At the end of the experiment (11 weeks), HFD-fed sedentary rats had increased body weight (Fig. 1A) and higher epididymal fat accumulation, developing hallmark characteristics of metabolic syndrome, such as significantly elevated fasting insulin levels and HOMA-IR and slight dyslipidemia [high-density lipoprotein (HDL), −15%; *P*<0.05], in comparison with age-matched control-diet-fed sedentary rats. No differences among groups were detected when measuring fasting blood glucose and low-density lipoprotein (LDL) cholesterol (Table 1). Conversely, 5-week-trained rats showed a lower final body weight than their counterparts in their respective diet groups and improved fasting insulin and insulin sensitivity (Fig. 1A, Table 1). A lower plasma leptin concentration in trained groups was accompanied by reduced appetite as a consequence of exercise performance (Table 1, Fig. 1B). Liver specimens from sedentary HFD-fed rats were paler, softer and had higher fat content than those from untrained rats fed a control diet or trained HFD-fed animals (Fig. 1C). Accordingly, sedentary HFD-fed rats exhibited microvesicular and macrovesicular liver steatosis, resulting in a significantly increased NAFLD activity score (NAS), while the hepatic histology from trained HFD-fed rats indicated reduced presence of hepatic lipid droplets (Fig. 1C,D). Although the hepatic histological changes induced by exercise might be considered to be modest, the benefits of exercise performance were supported by the observed improvement in the additional markers used to evaluate liver function. Thus, training significantly improved HFD-induced liver damage [alanine aminotransferase (ALT), −25%; aspartate aminotransferase (AST), −16%; lactate dehydrogenase (LDH), −33%; versus HFD, *P*<0.05], and tended to restore HFD-lowered plasma albumin levels (Table 1), indicating an important intrahepatic triglyceride-lowering potential (Fig. 1E). Altogether, these results endorse the protective effects of exercise performance on the HFD-induced disrupted metabolic status of NAFLD in young rats.

**Table 1.**
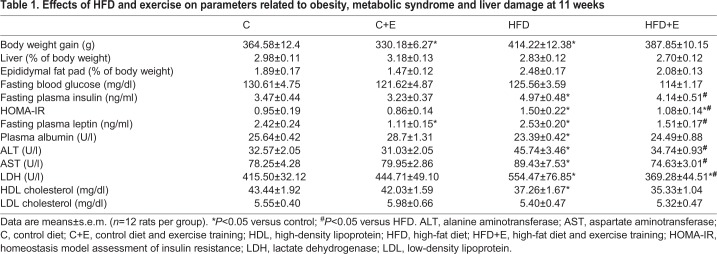
**Effects of HFD and exercise on parameters related to obesity, metabolic syndrome and liver damage at 11 weeks**

### Exercise exerts a positive modulatory effect on HFD-induced disrupted lipid metabolism

HFD-induced intrahepatic lipid accumulation appeared to be caused by altered lipid-metabolism-related gene expression. Consequently, the hepatic expression of crucial genes involved in *de novo* fatty acid (FA) synthesis, including liver X receptor alpha (*LXRα*; also known as *Nr1h3*), sterol regulatory element binding protein 1c (*SREBP-1c*; also known as *Srebf1*) and fatty acid synthase (*Fas*), as well as those involved in fatty acid uptake and transport, such as fatty acid translocase (*FAT*)/*Cd36*, were significantly upregulated after HFD intake (Fig. 1F). Accordingly, the mRNA expression level of their transcription factor regulator, CCAAT/enhancer-binding protein alpha (*C/EBPα*; also known as *Cebpa*), appeared significantly induced by HFD feeding. Conversely, exercise intervention effectively attenuated the hepatic gene deregulation induced by HFD. Moreover, lipid metabolism was also modified by exercise training in control-diet-fed rats, downregulating lipid-metabolism-related genes (*SREBP-1c*, *FAT*/*Cd36* and *C/EBPα*) (Fig. 1F).

### Effects of exercise and age on gut microbiota composition and short-chain fatty acid production in the early obesity and NAFLD model

**Fig. 2.**
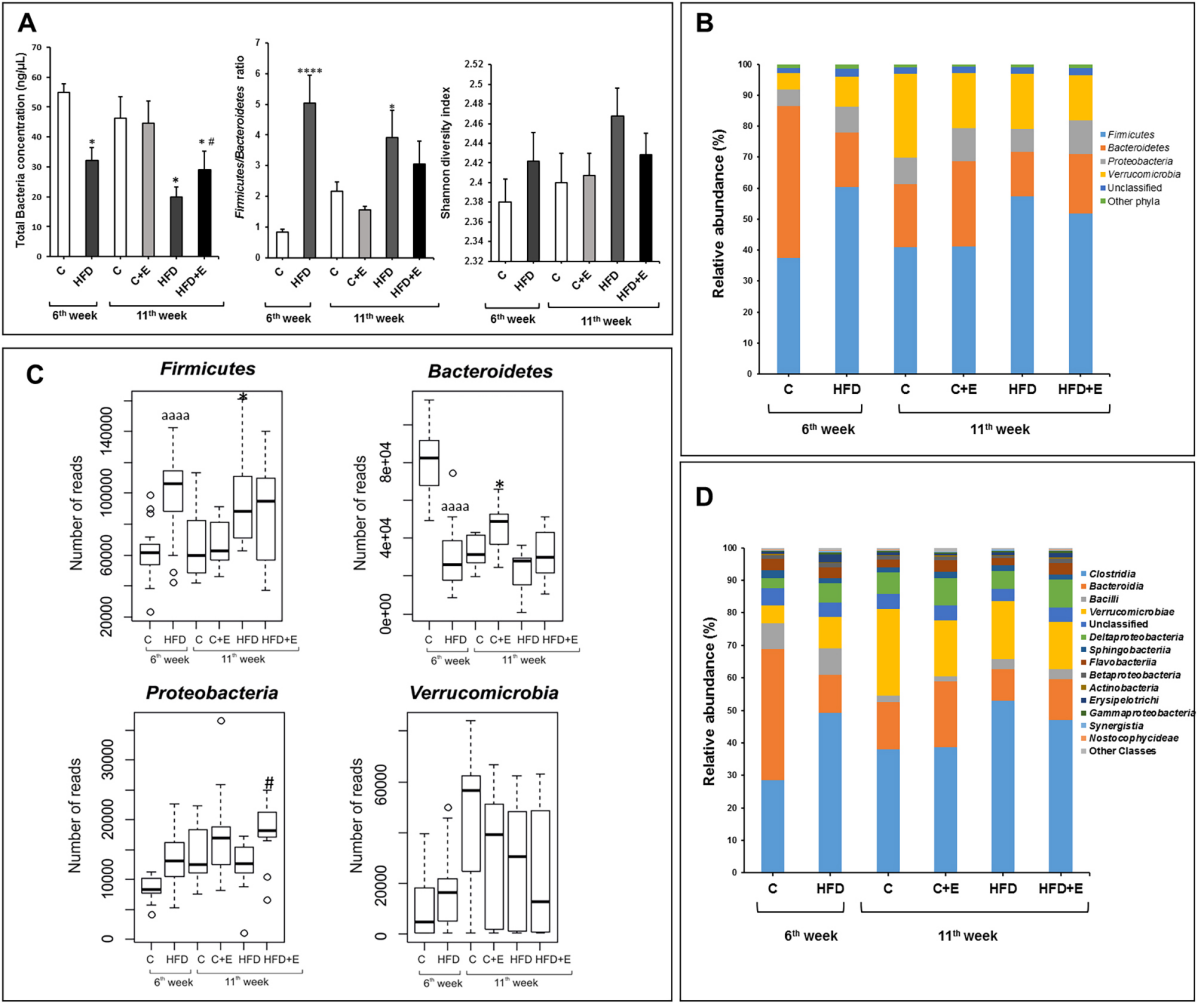
**Effects of diet, exercise and age on gut microbiota composition.** (A) Total bacterial concentration analyzed by quantitative PCR, Shannon diversity index and *Firmicutes*/*Bacteroidetes* ratio. Data are means±s.e.m. *P<0.05 versus control, ****P<0.0001 versus control; ^#^*P*<0.05 versus HFD. (B) Relative abundances of the indicated total populations at phylum level. (C) Box plots showing differences in the numbers of reads of *Firmicutes*, *Bacteroidetes*, *Proteobacteria* and *Verrucomicrobia* phyla. The boxes represent the interquartile range (IQR) between the first and third quartiles (25th and 75th percentiles, respectively) and the horizontal line inside the box defines the median. Whiskers represent the lowest and highest values within 1.5 times the IQR from the first and third quartiles, respectively. Samples exceeding those values are represented as points beside the boxes. Statistical analysis was performed using Kruskal–Wallis followed by Mann–Whitney *U*-test; ^aaaa^*P*<0.0001 versus control (week** **6); *P<0.05 versus control; ^#^*P*<0.05 versus HFD. (D) Relative abundances of the indicated populations at class level.

HFD intake resulted in a reduction in the total bacteria DNA concentration, but increased microbiota diversity, calculated by the Shannon diversity index (Fig. 2A). Nevertheless, exercise increased total bacteria DNA concentration and reduced the diversity index, to levels tending to become similar to those of the control group. A total of 14,251,285 high-quality sequences (reads) were obtained by high-throughput sequencing, ranging from 91,867 to 569,914 reads per sample before normalization by total number of reads. After 6 weeks, diet significantly modified the microbiota profile of pubertal rats at a phylum level. Thus, HFD intake increased the relative abundance of *Firmicutes* and lowered *Bacteroidetes* phyla, resulting in a significantly greater *Firmicutes*/*Bacteroidetes* ratio with regard to control-diet-fed rats (Fig. 2A), in agreement with previous results reported in obesity studies (Cheng et al., 2018; Porras et al., 2017). Similar ratios were maintained in sedentary rats until the end of the study (11 weeks). Exercise performance counteracted the HFD-induced microbial imbalance, reducing the *Firmicutes* abundance and increasing the relative presence of *Bacteroidetes*, thus modifying the *Firmicutes*/*Bacteroidetes* ratio (Fig. 2A)*.* Moreover, statistical analysis revealed that the relative abundance of *Bacteroidetes* and *Proteobacteria* phyla was significantly higher in the control-diet-fed, exercise-trained (C+E) and HFD-fed, exercise-trained (HFD+E) groups, respectively, compared with the corresponding untrained group (Fig. 2B,C).

At week 11, the rats showed higher relative abundance of *Proteobacteria* and *Verrucomicrobia* phyla and lower values of *Bacteroidetes* than those observed at week 6 (Fig. 2B,C), suggesting that, independently of diet and exercise, age behaves as an important microbiota modulatory factor.

**Fig. 3.**
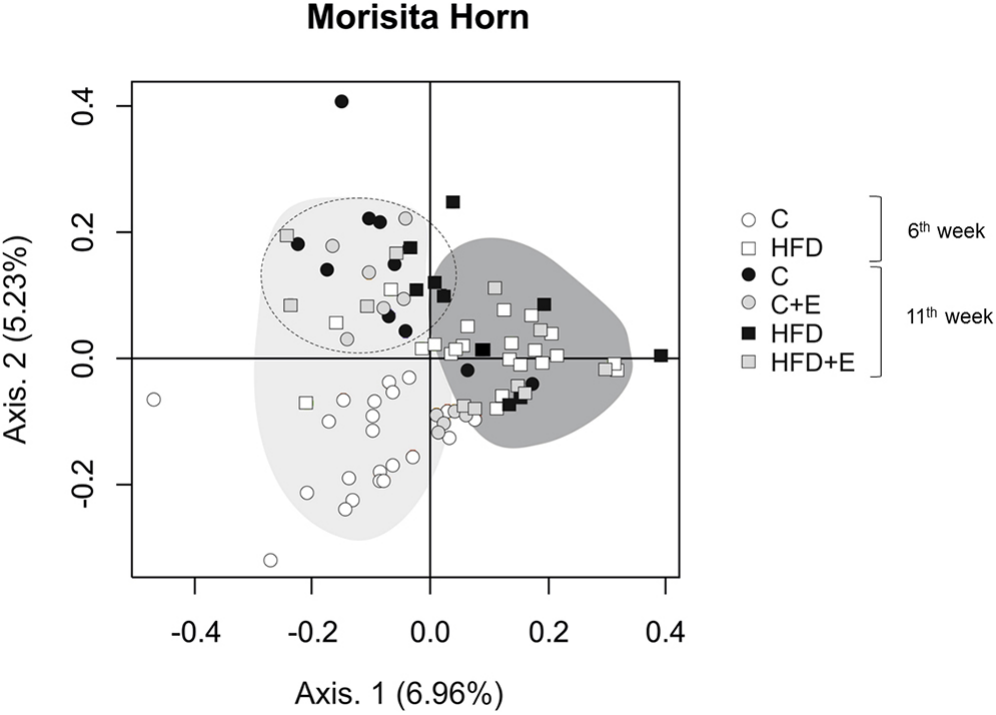
**Principal coordinates analysis (PCoA) plot derived from the Morisita-Horn dissimilarity index at the phylum level of the six experimental groups at 6 and 11 weeks.** The percentage of the total variance explained is indicated in parentheses in each axis. Shaded areas denote sample clusters according to diet (light gray, control; dark gray, HFD). The dashed line demarcates a subset associated with age.

Principal coordinates analysis (PCoA), based on the Morisita-Horn index, was performed to analyze the influence of age, diet and exercise on microbiota distribution at the phylum level. The first axis score plot (6.96%) revealed a clear separation of bacterial communities according to diet (*F*=10.299, *P*=0.0003) (Fig. 3). Conversely, exercise behaved as a dispersing factor (*F*=2.722, *P*=0.076). However, the second axis (5.23%) showed an age-specific pattern (*F*=15.901, *P*=0.0001) (Fig. 3).

At the class level (Fig. 2D), *Clostridia* (*Firmicutes* phylum), *Deltaproteobacteria* and *Gammaproteobacteria* (*Proteobacteria* phylum) were considerably increased in HFD-fed rats compared with control rats at week 6, whereas *Bacteroidia* (*Bacteroidetes* phylum) and *Bacilli* (*Firmicutes* phylum) were significantly reduced in the HFD group in comparison to the control group. Exercise training in HFD-fed rats substantially increased the relative abundance of *Deltaproteobacteria* and *Betaproteobacteria* (*Proteobacteria* phylum). In the control group, the relative abundance of *Bacilli*, *Verrucomicrobiae* (*Verrucomicrobia* phylum) and *Erysipelotrichi* (*Firmicutes* phylum) was significantly reduced after exercise training. *Bacteroidia*, *Bacilli* and *Sphingobacteriia* (*Bacteroidetes* phylum) were significantly reduced at the end of the study independently of diet, whereas *Deltaproteobacteria*, *Verrucomicrobiae* and *Gammaproteobacteria* showed an opposite pattern (Fig. 2D), revealing an age-dependent contribution to the bacteriome profile at the class taxa.

**Fig. 4.**
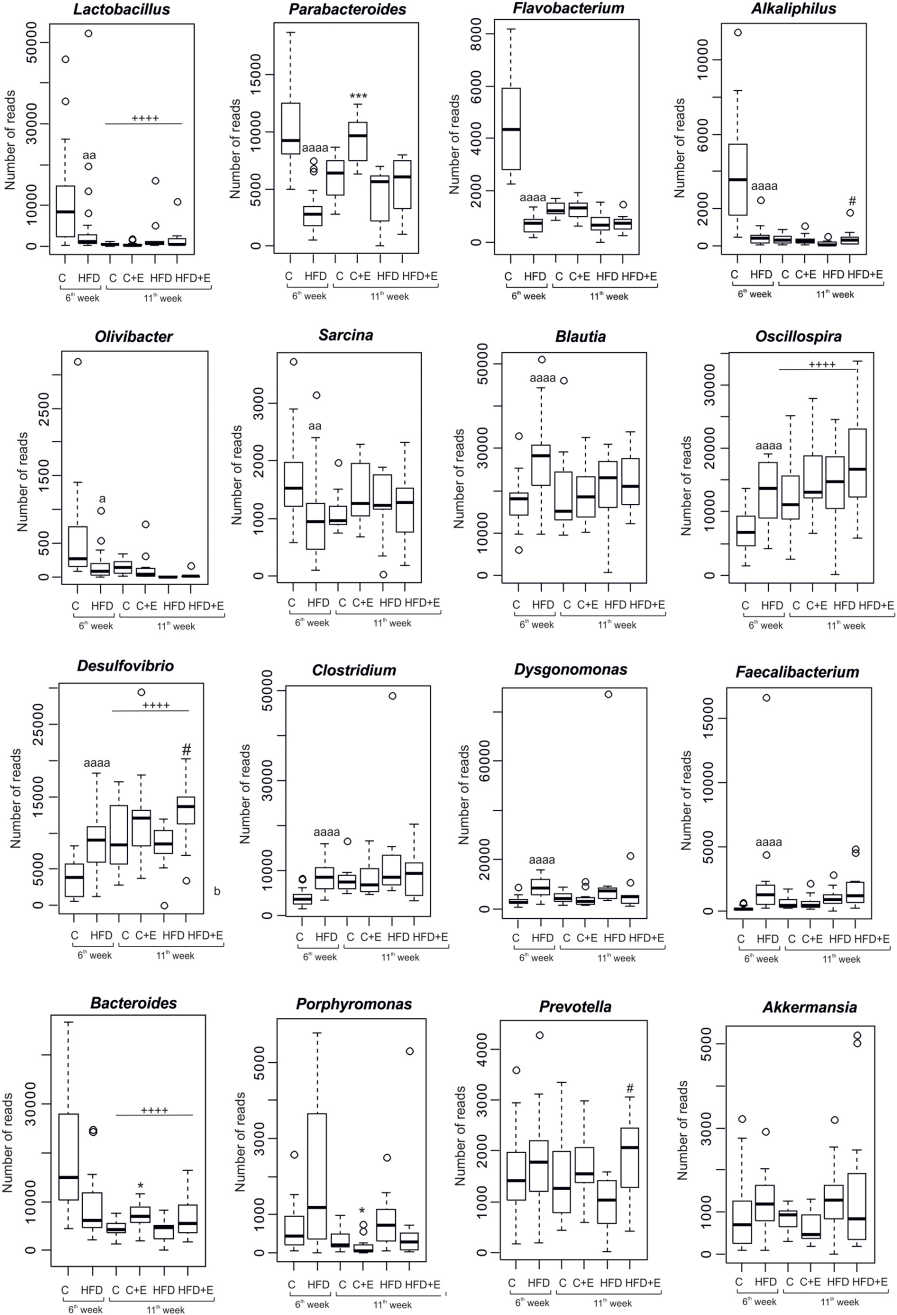
**Effects of diet, exercise and age on gut microbiota balance at genus level.** Box plots representing the differences between control and HFD-fed rats with and without exercise training at 6 and 11** **weeks. The boxes represent the interquartile range (IQR) between the first and third quartiles (25th and 75th percentiles, respectively) and the horizontal line inside the box defines the median. Whiskers represent the lowest and highest values within 1.5 times the IQR from the first and third quartiles, respectively. Samples exceeding those values are represented as points beside the boxes. Statistical analysis was performed using the Mann–Whitney *U*-test; ^a^*P*<0.05, ^aa^*P*<0.01, ^aaaa^*P*<0.0001 versus control (6** **weeks); *P<0.05, ***P<0.001 versus control; ^#^*P*<0.05 versus HFD; ^++++^*P*<0.0001 versus 6** **weeks, independently of diet. *P*-values are corrected for multiple comparisons based on false discovery rate (FDR).

Accordingly, diet, age and exercise also modified the rat gut microbiota at genus level (Fig. 4). After 6 weeks, HFD significantly reduced the relative abundance of *Lactobacillus*, *Parabacteroides*, *Flavobacterium*, *Alkaliphilus*, *Olivibacter* and *Sarcina*, and increased the read numbers of *Blautia*, *Oscillospira*, *Desulfovibrio*, *Clostridium*, *Dysgonomonas* and *Faecalibacterium* genera, compared with those observed after the control diet (Fig. 4). Exercise substantially corrected HFD-induced microbial imbalance, and modified the relative abundance of *Parabacteroides*, *Clostridium*, *Flavobacterium* and *Alkaliphilus*, to levels that were similar to those found in control sedentary rats.

The training effects were different depending on the diet. In exercise-trained rats fed with control diet, higher levels of *Parabacteroides* and *Bacteroides* were detected compared with the corresponding sedentary group (Fig. 4). A different profile was found in HFD-fed trained rats, which showed significant changes in the amount of *Desulfovibrio*, *Prevotella* and *Alkaliphilus*. Independently of diet, rats at 11 weeks presented with higher relative abundance of *Oscillospira* and *Desulfovibrio*, and reduced presence of *Lactobacillus* and *Bacteroides*, in comparison to that observed 5 weeks earlier (Fig. 4), highlighting the importance of age in defining the microbiota profile.

Disturbed fecal production of short-chain fatty acids (SCFAs) has been described as being related to changes in microbiota composition (Porras et al., 2018). Similarly, in our study, HFD intake after 6 weeks was associated with significantly lower acetate and propionate production in feces. At week 11, exercise in control-diet-fed rats tended to increase the production of SCFAs associated with gut microbiota composition; this effect was attenuated when rats were fed an HFD (Fig. S3).

### Correlation between gut microbiota and fecal metabolome in our *in vivo* model of early obesity and NAFLD

**Fig. 5.**
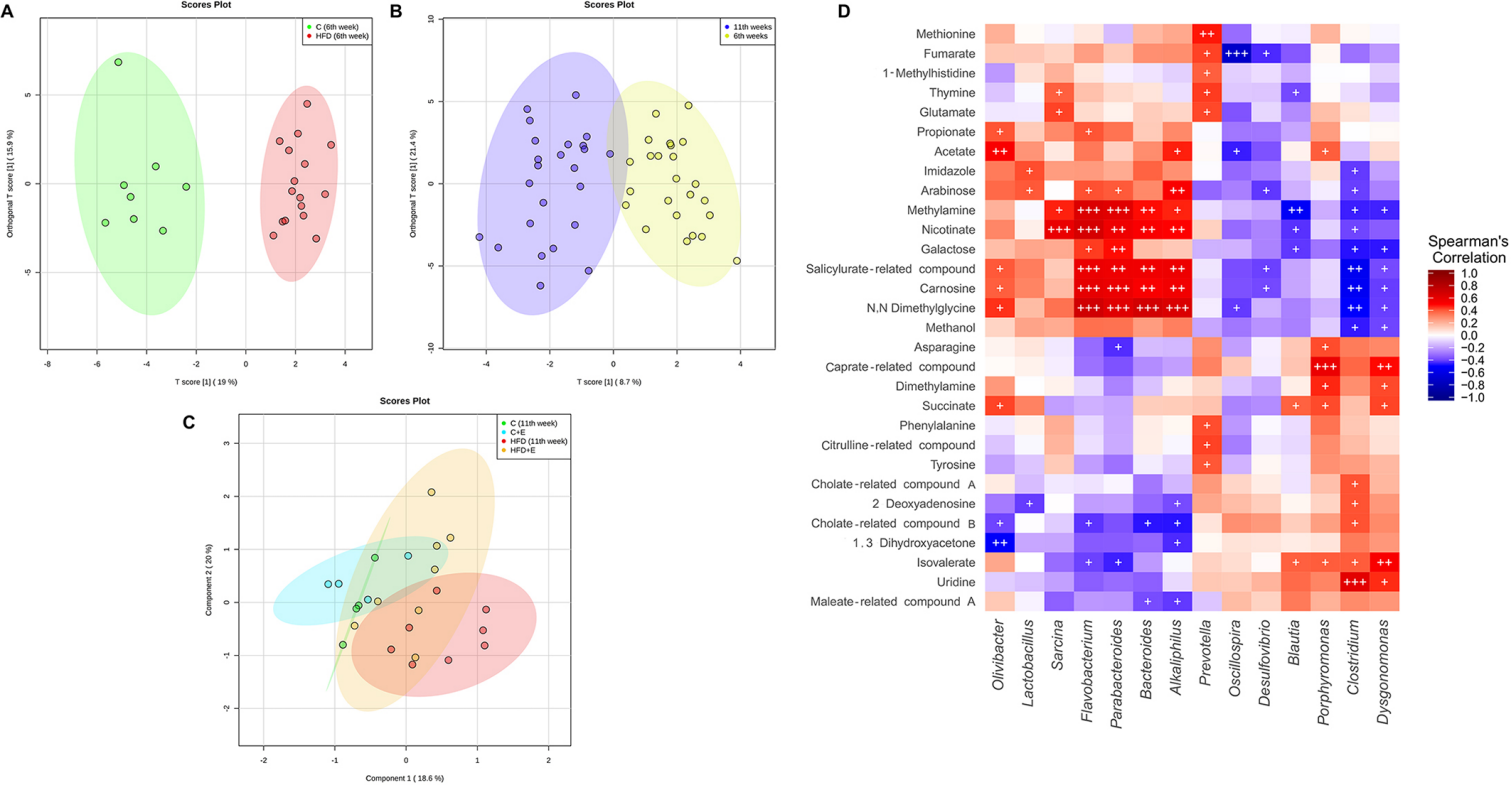
**Relationship between gut microbiota and fecal metabolome.** (A) Orthogonal projections to latent structures discriminant analysis (OPLS-DA) of metabolites from control and HFD-fed rats at 6** **weeks. (B) OPLS-DA showing the effect of age on metabolomic profile. (C) Partial least squares discriminant analysis (PLS-DA) of metabolites from control and HFD-fed rats with and without exercise training at 11 weeks. Colored ellipses represent the 95% confidence range for the indicated experimental group. (D) Spearman's correlation analysis was used to investigate the relationship between fecal bacterial populations and metabolite levels, considering results obtained longitudinally in all groups. Red and blue cells indicate positive and negative correlations, respectively. ^+^*P*<0.05, ^++^*P*<0.01, ^+++^*P*<0.001. *P*-values are corrected for multiple comparisons based on FDR.

A total of 114 fecal metabolites were detected in all groups, including BAs, SCFAs, free fatty acids, lipids, amino acids, carbohydrates, nucleotides and organic acids. The orthogonal projections to latent structures discriminant analysis (OPLS-DA) score plot revealed a manifest separation between control and HFD-fed rats at week 6 according to diet (Fig. 5A). Moreover, a clear age-specific pattern associated with metabolic profile was shown independently of diet (Fig. 5B). At week 11, according to partial least squares discriminant analysis (PLS-DA) score, HFD-fed sedentary rats and control-diet-fed rats still clustered separately; however, exercise acted as a dispersing factor, so HFD-fed trained rats formed a heterogeneous group between the control and HFD animals (Fig. 5C).

Functionality correlations between the differential gut microbiota composition and the fecal metabolites were performed, considering results obtained longitudinally in all experimental groups. The Spearman's correlation coefficient (r) was computed for 78 different metabolites and 16 bacterial taxa (Fig. 5D). The results showed 104 statistically significant interactions between 30 metabolites and 14 bacterial taxa (*P*<0.05). The presence of *Parabacteroides*, *Flavobacterium* and *Alkaliphilus* genera, reduced by HFD feeding and increased by exercise training, had a strong positive correlation with the fecal metabolites N,N-dimethylglycine, carnosine, salicylurate-related compound, nicotinate, methylamine, trimethylamine and arabinose. A similar metabolic profile was associated with *Bacteroides*, which was also reduced by HFD feeding and increased by exercise training (Fig. 5D), whereas the presence of the *Olivibacter* genus, decreased in HFD-fed rats, positively correlated with acetate and propionate. Interestingly, most of these metabolites negatively correlated with *Clostridium* and *Dysgonomonas* genera, both of which were increased in HFD-fed rats and decreased by exercise training. Presence of *Blautia* also negatively correlated with methylamine and nicotinate, appeared enriched as a result of HFD feeding and was reduced by exercise. HFD-dependent *Desulfovibrio* presence negatively correlated with carnosine and salicylurate-related compound (Fig. 5D).

Another gut metabolome pattern was also associated with some bacterial genera increased by HFD feeding. Isovalerate, succinate, dimethylamine and caprate-related compound positively correlated with *Dysgonomonas* and *Porphyromonas* genera. Moreover, *Dysgonomonas* and *Porphyromonas* positively correlated with uridine and asparagine, respectively (Fig. 5D). An altered BA metabolism was associated with *Clostridium* genus, which was significantly increased by HFD feeding, showing a positive correlation with cholate-related compounds. Additionally, this genus was also positively associated with uridine, 2-deoxyadenosine and isovalerate presence in feces. By contrast, cholate-related compound, isovalerate, 2-deoxyadenosine, maleate-related compound and 1,3-dihydroxyacetone negatively correlated with *Alkaliphilus*, *Flavobacterium*, *Bacteroides* and *Parabacteroides*, bacterial genera diminished in HFD-fed juvenile rats (Fig. 5D).

Interestingly, *Prevotella*, increased in the HFD-fed trained group compared with its respective sedentary group, presented an associated specific metabolomic profile, showing a positive significant correlation with thymine, methionine, 1-methylhistidine, fumarate, glutamate, cadaverine, tyrosine, citrulline-related compound and phenylalanine, predominantly amino-acid-related metabolites (Fig. 5D).

### Exercise improves HFD-mediated barrier disruption, and counteracts endotoxemia, oxidative stress, activation of the gut-liver axis and inflammatory response in our *in vivo* model of early obesity and NAFLD

**Fig. 6.**
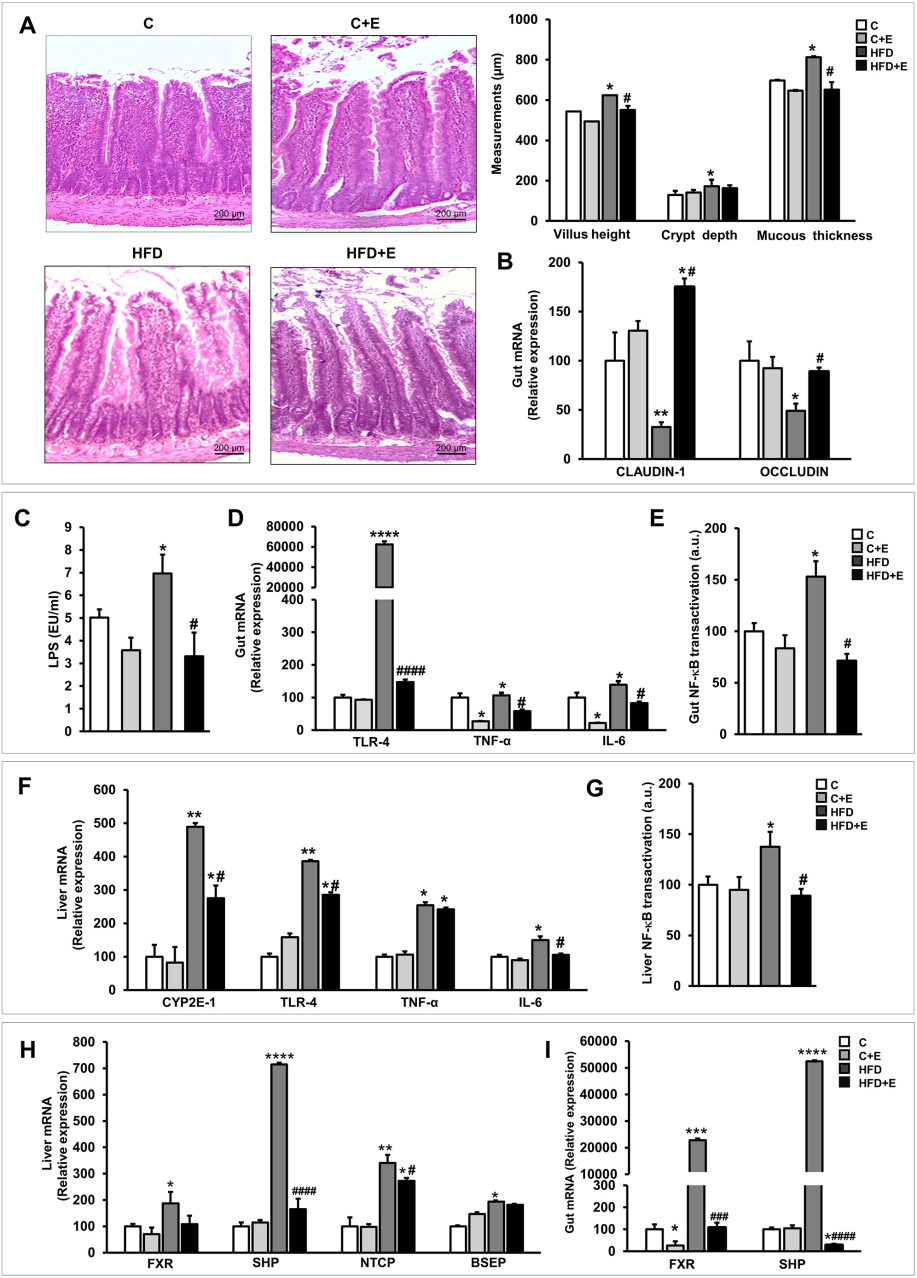
**Effects of diet and exercise intervention on intestinal barrier integrity, activation of the gut-liver axis and bile acid (BA)-metabolism-related gene expression in control and HFD-fed rats at 11 weeks.** (A) Representative H&E-stained small intestine sections (left). Scale bar: 200 μm. Bar graph showing villus height, crypt depth and mucosa thickness linear measurements (right). (B) mRNA levels of intestinal claudin-1 and occluding, determined by RT-qPCR. (C) Plasma LPS levels were measured using an LAL Chromogenic Endotoxin Quantitation Kit. (D,F) mRNA levels of *Cyp2e1*, *Tlr-4*, *Tnf-a* and *Il-6* (F, liver samples; D, gut samples). (E,G) NF-κB (p65) transcriptional activity (G, liver samples; E, gut samples). (H,I) mRNA levels of BA-metabolism-related genes (H, liver samples; I, gut samples). Data are means±s.e.m. (*n*=12 rats per group). *P<0.05, **P<0.01, ***P<0.001, ****P<0.0001 versus control; ^#^*P*<0.05, ^###^*P*<0.001, ^####^*P*<0.0001 versus HFD.

In response to HFD, juvenile rats showed irregularly arranged microvilli, higher villus height and crypt depth, and increased mucosa thickness (Fig. 6A). Alterations in tight junctions and epithelial adhesion molecules can increase the intestinal barrier permeability, allowing the influx of immune-stimulatory microbial metabolites across the epithelial layer, leading to mucosal infections commonly observed in obese/NAFLD patients (Thaiss et al., 2018). Tight-junction integrity was compromised in sedentary HFD-fed rats, revealed by the underexpression of the intestinal genes claudin-1 and occludin, directly related to leaky epithelia (Fig. 6B). In line with these results, HFD feeding enhanced lipopolysaccharide (LPS) influx across the gastrointestinal epithelium (Fig. 6C). Conversely, aerobic exercise performance improved intestinal mucosa morphology, and resulted in the upregulation of claudin-1 and occludin mRNA levels, accompanied by reduced endotoxemia (Fig. 6A-C), supporting the capacity of exercise training to partially restore intestinal barrier function in our experimental model of early obesity and NAFLD.

The flux of fatty acids through the liver was increased in our HFD-induced NAFLD model, likely as a consequence of inappropriate lipid metabolism, with associated imbalanced fatty acid oxidation. These changes were accompanied by dramatically enhanced hepatic *Cyp2e1* expression, involved in oxidative stress development and derived lipotoxicity. Exercise performance caused a distinct reduction in HFD-induced hepatic *Cyp2e1* expression (Fig. 6F).

HFD-induced endotoxemia was accompanied by a marked *Tlr-4* gene upregulation, enhanced NF-κB (also known as Rela) transactivation and subsequent inflammation in both liver and gut (Fig. 6D-G). Conversely, exercise performance effectively counteracted HFD-induced activation of the gut-liver axis, lowering hepatic and intestinal *Tlr-4* mRNA levels, and preventing NF-κB-dependent inflammatory response, as evidenced by the reduced hepatic and intestinal mRNA levels of *Tnf-a* and *Il-6* (Fig. 6D-G).

### Exercise improves HFD-induced obesity and hepatic steatosis through its capacity to modulate BA metabolism and enterohepatic circulation

Feeding rats a HFD deregulated hepatic BA uptake, significantly upregulating the expression of Na^+^ taurocholate co-transporter (*NTCP*; also known as *Slc10a1*) and bile salt export pump (*BSEP*; also known as *Abcb11*). These effects were linked to enhanced nuclear farnesoid-X-receptor (*FXR*; also known as *Nr1h4*) hepatic expression, leading to dramatic induction of the small heterodimer partner (*SHP*; also known as *Nr0b2*) (Fig. 6H). HFD-induced impaired enterohepatic BA recycling was accompanied by an altered fecal BAs metabolome profile, in a mechanism involving microbiota composition and functionality disturbance. The findings concerning fecal BA-related metabolome might be a cause or a consequence of the robust FXR-SHP signaling activation detected in HFD-fed rat small intestine, where *FXR* appeared upregulated, causing increased *SHP* expression (Fig. 6I). Interestingly, daily exercise performance restored HFD-induced hepatic and intestinal *FXR* expression levels to those observed in sedentary control rats, which completely abrogated hepatic and intestinal *SHP* gene expression (Fig. 6H,I), and similarly counteracted the HFD-mediated *NTCP* and *BSEP* hepatic overexpression (Fig. 6H).

### Correlation between gut microbiota composition and early-obesity-associated NAFLD spectrum

**Fig. 7.**
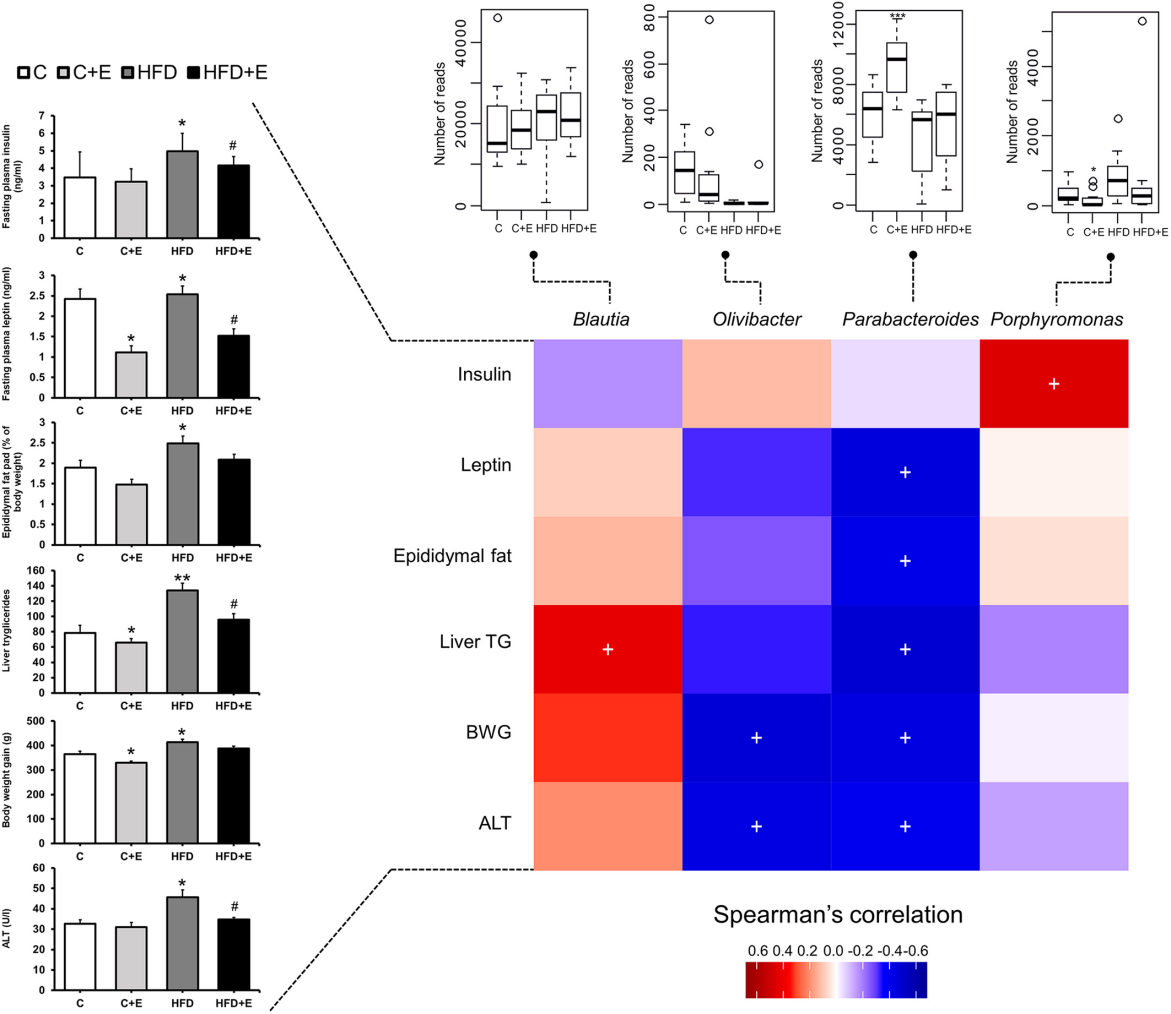
**Correlation between gut microbiota composition and early-obesity-associated NAFLD spectrum.** Spearman's coefficient (*r*), ranging from positive (red) to negative (blue) values, was used to cross-correlate bacterial genera and phenotypic parameters associated with early obesity and NAFLD at 11** **weeks. ^+^*P*<0.05. Box plots represent the differences between control and HFD-fed rats with and without exercise training at week 11 of the study. The boxes represent the interquartile range (IQR) between the first and third quartiles (25th and 75th percentiles, respectively) and the horizontal line inside the box defines the median. Whiskers represent the lowest and highest values within 1.5 times the IQR from the first and third quartiles, respectively. Samples exceeding those values are represented as points beside the boxes. Fasting insulin and leptin levels, epididymal fat (as % of body weight), liver triglycerides, body weight gain and ALT levels at week 11 are represented as bar graphs. *P<0.05, **P<0.01, ***P<0.001 versus control, ^#^*P*<0.05 versus HFD.

The relative abundance of *Parabacteroides* was negatively correlated with body weight gain (*P*<0.05), hepatic triglyceride content (*P*<0.05), epididymal fat (*P*<0.05), ALT (*P*<0.05) and leptin (*P*<0.05) plasma levels in our early obesity and NAFLD model. The *Olivibacter* genus was also negatively correlated with body weight gain (*P*<0.05) and ALT plasma levels (*P*<0.05). *Blautia* and *Porphyromonas* presence showed a positive correlation with hepatic triglyceride content (*P*<0.05) and insulin levels (*P*<0.05), respectively (Fig. 7).

## DISCUSSION

NAFLD prevalence in the pediatric population has increased dramatically, being the major cause of liver failure and indication for liver transplantation in childhood and adolescence in the Western world (Giorgio et al., 2013; Mathur et al., 2007). First-line NAFLD interventions focus on reducing the etiology-related factors, obesity and insulin resistance, through diet and increased physical activity (Mathur et al., 2007). Thus, intrinsic aerobic capacity determines susceptibility to HFD-induced hepatic steatosis, as a consequence of different metabolic functionality of the gut microbiota (Matthew Morris et al., 2014; Panasevich et al., 2016). Accordingly, the benefits of different types of exercise protocols on metabolic syndrome have been widely reported, with a clear dose-response association (Ordoñez et al., 2015). We developed a novel and reliable nutritional model of juvenile obesity-related NAFLD to evaluate and characterize the molecular mechanisms underlying the effects of exercise intervention on gut microbiota composition and functionality, and consequent effects on early obesity and NAFLD onset in pediatric patients.

To our knowledge, this is the first study using HFD-fed 21-day-old rats to simulate the NAFLD disease occurring in pediatric patients; nonetheless, similar age juvenile rats have previously been chosen to reflect obesity-related disorders and other pediatric pathologies (Botezelli et al., 2010; Holl et al., 2018). The use of a pubertal model of NAFLD is of particular relevance because insulin or glucose levels have been reported not to differ between pre-pubertal children with or without NAFLD, whereas HOMA-IR or oral glucose tolerance test results change in adolescents (Ozhan et al., 2015). Therefore, understanding how early intervention might affect long-term disease that is specific to this age range in this model would be of interest. In our study, HFD-fed rats displayed metabolic-syndrome-related characteristics, increased body weight gain and fat deposition, and impaired liver function and steatosis, key hallmarks of the NAFLD phenotype observed in children (Mathur et al., 2007), corroborating the suitability of our NAFLD model.

As previously mentioned, exercise practice is prescribed for NAFLD patients (Zhang et al., 2016). In this regard, Coll-Risco et al. (2016) indicated that moderate aerobic training improved body composition and metabolic status in obese rats, encouraging us to further explore the effects of this exercise protocol in our *in vivo* model. In our study, HFD-fed trained juvenile rats experienced a reduction of appetite and a ∼7% weight loss closely linked to the substantial improvement in NAFLD outcomes, as previously reported in *in vivo* models and clinical trials in adults (Kapravelou et al., 2015; Romero-Gómez et al., 2017).

Although the benefits of exercise prescription for NAFLD patients are widely documented, the underlying molecular mechanisms remain unknown. Our results suggest that exercise training improves HFD-induced hepatic steatosis by restoring disrupted lipid-metabolism homeostasis, attenuating *de novo* fatty acid synthesis and uptake-related gene upregulation, as well as diminishing *CYP2E1* overexpression, which can also impact lipid homeostasis (Aubert et al., 2011), closely associated with NAFLD progression, as previously described in adult patients (Mitsuyoshi et al., 2009). Supporting our results, exercise protocols in rodent models of NAFLD have shown their capacity to counteract lipid-metabolism deregulation (Linden et al., 2013, 2014). Moreover, earlier meta-analysis indicates that different forms of aerobic, resistance or high-intensity intermittent exercise improve NAFLD outcomes (Keating et al., 2012).

Some interesting findings concerning the effects of training on control animals, in which, similarly to the HFD exercised group, there was a decrease in appetite that could be linked, in both cases, to the effects of chronic exercise on plasma leptin levels already described in rodents and human (Fedewa et al., 2018; Zheng and Niu, 2018). Moreover, we report – for the first time – that trained animals showed a decrease in the expression of liver *SREBP-1c*, *FAT*/*Cd36* and *C/EBPα*, supporting the contribution of lipogenesis to the effect of exercise on hepatic fat accumulation (Yoshimura et al., 2018).

The relationship between obesity and altered microbiota has been widely described in rodents and patients (Del Chierico et al., 2017; Porras et al., 2017). Moreover, HFD feeding causes gut microbiota imbalance associated with obesity-related NAFLD development, justifying the current study (Kucera and Cervinkova, 2014; Porras et al., 2017). Our metagenomic analyses showed an altered gut microbiota profile at phylum, class and genus levels in HFD-fed juvenile rats, leading to microbial imbalance. HFD intake reduced bacteria concentration and diversity, increasing the *Firmicutes*/*Bacteroidetes* ratio in comparison to that of the control group, as previously described (Cheng et al., 2018; Porras et al., 2017). The influence of age on gut microbiota has also been reported in pediatric-obesity-related NAFLD patients (Del Chierico et al., 2017). We found age-specific microbiota composition and associated metabolomic profile patterns along the study. In addition, HFD-induced altered production of SCFAs improved with age, suggesting a possible age-dependent association between obesity-related NAFLD and microbiota diversity and functionality.

Recent research highlights the capacity of exercise to impact the diversity, composition and functionality of gut microbial populations in *in vivo* models of obesity (Evans et al., 2014; Lai et al., 2018; Petriz et al., 2014) and human studies (Cronin et al., 2018; Zhao et al., 2018). In our study, exercise intervention balanced HFD-induced alterations in microbiota composition, promoting opposite changes in fecal microbiota to those characteristic of obesity and related metabolic alterations. Furthermore, HFD feeding reduced the relative abundance of *Parabacteroides*, *Flavobacterium* and *Alkaliphilus* genera, which appeared normalized upon exercise performance. Interestingly, these genera could be associated with a protective effect against early obesity and NAFLD. In this regard, the relative abundance of *Parabacteroides*, a genus predominantly found in the gut of healthy individuals (Xu et al., 2007), negatively correlated with body weight gain, liver steatosis and damage, epididymal fat accumulation and leptin plasma levels. Lower detection of *Flavobacterium* and *Alkaliphilus* genera also appeared in our previous NAFLD nutritional model in adult mice, related to increased susceptibility to NAFLD development (Porras et al., 2017; Porras et al., 2019).

Furthermore, *Parabacteroides*, *Flavobacterium* and *Alkaliphilus* genera positively correlated with fecal abundance of carnosine, nicotinate, methylamine and arabinose metabolites. Altered nicotinate and methylamine metabolism, reduced in HFD-fed juvenile rat feces, are linked to obesity and diabetes (Hao et al., 2015; Palau-Rodriguez et al., 2015; Pelantová et al., 2016; Schaalan et al., 2018). Studies have reported the anti-obesity and anti-inflammatory capacities of carnosine and arabinose, which are promising candidates for therapies combating obesity, metabolic syndrome and NAFLD (Hao et al., 2015; Schaalan et al., 2018). Another potentially protective genus was *Olivibacter*, also showing lower detection in HFD-fed compared with control animals. Thus, this genus negatively correlated with body weight gain and ALT plasma levels, in a mechanism that could involve its positive association with fecal acetate and propionate metabolites, supporting the protective role of SCFAs in gut microbiota functionality and barrier integrity previously described (Canfora et al., 2017). Other genera such as *Blautia*, *Porphyromonas*, *Dysgonomonas* and *Clostridium* exhibited an opposite pattern, with higher detection in HFD-fed juvenile rats, which was partially reverted by exercise intervention. Higher presence of *Faecalibacterium* genus was also detected in HFD-fed juvenile rats; however, the levels of *Faecalibacterium* *prausnitzii* have been found to be decreased in adult patients suffering from obesity and metabolic disorder (Furet et al., 2010). A recent study has shown that the *Blautia* genus appears to be increased in patients with obesity and NAFLD (Shen et al., 2017). Moreover, our results showed a positive correlation between this genus and hepatic triglyceride accumulation, suggesting its possible involvement in early-obesity-associated NAFLD onset. Additionally, increased presence of the *Blautia* genus was associated with a specific metabolic profile characterized by restricted methylamine and nicotinate presence in feces. *Porphyromonas* genus, also enriched as a result of HFD feeding, has been associated with inflammatory pathologies (Carter et al., 2017). In our research, this genus positively correlated with insulin levels and fecal metabolites, such as asparagine, succinate and dimethylamine. Increased presence of these metabolites in the feces of patients with obesity-related diseases has been described in several studies (Reverri et al., 2017; Schlecht et al., 2017; Serena et al., 2018). Moreover, *Clostridium* genus was associated with a defined metabolic profile compatible with altered BA fecal metabolome, showing a positive correlation with cholate-related compound presence in feces, contrasting with the opposite profile observed in HFD-inhibited genera. Likewise, Zheng et al. (2017) reported HFD-induced impaired enterohepatic BA recycling. In this regard, exercise performance effectively restored HFD-induced impaired BA metabolism, reducing BA transporter upregulation, *FXR* induction and subsequent *SHP* overexpression in liver and small intestine. It has been described that activation of the FXR-dependent pathway promotes metabolic disease in diet-induced and genetically obese rodents in a mechanism involving microbial imbalance (Gonzalez et al., 2016), due to the capacity of intestinal microbiota to alter the BA pool and signaling properties (Arab et al., 2017). Thus, exercise could influence BA metabolism through its ability to modulate gut microbiota diversity and functionality. The effects of training on the microbiota differed according to the diet source. Interestingly, the combination of exercise and HFD resulted in a significant increase in the relative abundance of *Prevotella* genus, which positively correlated with amino-acid-related metabolites, as previously described in humans (Petersen et al., 2017).

In the multiple parallel hits hypothesis, Tilg and Moschen (2010) highlighted the importance of microbiota-associated alteration of the gut-liver axis due to compromised intestinal barrier integrity and functionality in NAFLD pathogenesis. In our juvenile rat model, HFD-induced changes in microbiota promoted intestinal barrier disruption and inflammatory and metabolic gene deregulation. Nonetheless, exercise performance effectively improved diet-induced morphological and functional alterations in juvenile rat small intestine, ameliorating altered intestinal barrier permeability and associated gut-liver crosstalk derangement. Thereby, exercise prevented LPS influx, leading to TLR-4-mediated NF-κB activation and downstream TNF-α and IL-6 production. Animal studies have revealed a tendency towards the downregulation of TLR-2 and TLR-4 after chronic exercise, accompanied by reduced activation of NF-κB and cytokine production and an improvement in insulin sensitivity (Rada et al., 2018). Current evidence in humans also suggests a role of TLRs in the anti-inflammatory properties of exercise (Rodriguez-Miguelez et al., 2014). Our data correlate with the capacity of exercise to maintain gut integrity and counteract both intestinal and systemic inflammatory response, even in the presence of an HFD (Campbell et al., 2016; Monda et al., 2017).

Together, our results suggest the existence of an HFD-determined deleterious microbiota profile that is positively modified by exercise intervention, leading to a functionally protective microbiota able to counterbalance altered intestinal barrier permeability, gut-liver axis and BA circulation. Thus, we provide a mechanistic understanding of the beneficial effects of physical exercise in pediatric obesity-related NAFLD patients, based on microbiota composition and functionality modulation, and reinforce the value of exercise performance as an efficient non-pharmacologic therapy.

## MATERIALS AND METHODS

### Animals and experimental diets

Juvenile (21-day-old) male Wistar rats were separated into two subgroups (*n*=24 each) based on their diet (Research Diets, New Brunswick, NJ, USA; Table S1): control (10% energy from fat; D12450J) and a semi-purified HFD (60% energy from fat; D12492). Six weeks of HFD feeding allowed the onset of obesity and metabolic syndrome features in pubertal rats. After this period, each diet group was split into two categories of equal average body weight, exercise or sedentary, based on whether the animals undertook the training protocol or not, for 5 additional weeks (*n*=12 each; Fig. S2). Throughout the experimental period, rats were housed under controlled conditions of temperature, humidity and lighting, had free access to water and consumed the diet *ad libitum*. Body weight and food intake were monitored weekly. All procedures were approved by the local Animal Ethics Committee and performed in accordance with the European Research Council guidelines for animal care and use.

### Exercise protocol

The exercised experimental group followed a combined aerobic and resistance training protocol modified from Coll-Risco et al. (2016). Training sessions consisted of 60 min of effective work, starting with a 10-min running warm up, followed by resistance training, consisting of eight 2-min running bouts (separated by 1 min of rest), during which the animals ran with an incline, which was progressively increased from 10° to 25° at a constant slow speed (20-25 cm/s). This exercise training was followed by 30 min of continuous aerobic exercise on the treadmill (Table S2). At the end of the experimental period (11 weeks), rats were euthanized with pentobarbital and bled by cannulation of the carotid artery 48 h after the exercise training.

### Sample collection

Plasma was isolated for biochemical determinations. Liver and epididymal white adipose tissue were collected and weighed on the precision balance. Subsequently, fecal samples, portions of liver, epididymal white adipose tissue, small intestine and vastus lateralis muscle were immediately collected, rinsed in saline solution, snap frozen in liquid nitrogen and stored at −80°C until further analysis. The right posterior lobe of the liver and a part of the gut were fixed in 10% formalin for histology. The remaining parts of the liver and gut were stored in RNAlater preservative at −80°C for further analysis.

### Histopathology

Formalin-fixed and paraffin-embedded liver and gut samples were sectioned and stained with Hematoxylin and Eosin (H&E). In the liver samples, hepatic lesions were evaluated by a histological scoring system for NAFLD proposed by Kleiner et al. (2005). The NAS was used as a tool to provide a numerical score evaluating three histological features semi-quantitatively: steatosis (0-3), lobular inflammation (0-3) and hepatocellular ballooning (0-2). Samples with scores more than 5 were correlated with a diagnosis of non-alcoholic steatohepatitis (NASH) and scores less than 3 were diagnosed as ‘not NASH’. From the gut sections, three 3-mm^3^ tissue pieces were fixed in 10% neutral buffered formalin, sectioned at 2.5 µm, stained with H&E, and viewed under a regular light microscope at 100× magnification. Ten villus height, crypt depth and mucosa thickness linear measurements were obtained. Evaluations were performed blinded to the sample source.

### Biochemical analysis

Plasma levels of ALT and AST, LDH activities and intrahepatic triglycerides were analyzed by the Instrumental Techniques Laboratory of the University of León using standard techniques. Plasma LPS quantification was performed using an LAL Chromogenic Endotoxin Quantification Kit (Thermo Fisher Scientific, Rockford, IL, USA), according to the manufacturer's instructions. Plasma levels of insulin and leptin were determined by specific enzyme-linked immunosorbent assay (ELISA) kits (Millipore, Darmstadt, Germany), according to the manufacturer's instructions. Plasma glucose levels were measured with an Accu-Chek (Roche Diagnostics, Almere, The Netherlands) after a 12-h fast. The HOMA-IR was used to calculate insulin resistance:

HOMA-IR=fasting glucose (mg/dl)×fasting insulin (μU/ml)/405.

Citrate synthase activity was determined spectrophotometrically from vastus lateralis muscle samples according to the methods of Faloona and Srere (1969). Values were then normalized to the total protein content quantified by bicinchoninic acid reagents (Pierce, Rockford, IL).

### Intestinal microbiota analysis

Genomic DNA was extracted from fecal samples using a DNA Stool Mini Kit (Qiagen, Hilden, Germany), with modifications to the manufacturer instructions. An initial bead-beating step was introduced to enhance homogenization, and the lysis temperature was increased up to 90°C to improve the DNA recovery yield. Amplification of the 16S rRNA V3-V4 hypervariable region was performed as previously described (Porras et al., 2017). The resulting amplicons were cleaned, quantified and sequenced on an Illumina MiSeq platform. Samples were analyzed using BaseSpace Application 16S Metagenomics v1.0 (Illumina Inc.). ‘Quantitative Insights into Microbial Ecology’ software (QIIME version 1.9.0; Caporaso et al., 2010) was used to verify the results. Processed reads were then clustered into operational taxonomic units (OTUs) using UCLUST with a similarity threshold of 0.97, and were subsequently aligned using PyNast against 16S reference database GreenGenes version 13.8 using default parameters. These OTUs were analyzed with the Vegan package in R software (https://cran.r-project.org/web/packages/vegan). Alpha and beta diversity were calculated and permutational multivariate analysis of variance (PERMANOVA) was used to determine the significance of the different factors’ effects on bacterial communities.

### Fecal metabolite profile assessment

Approximately 70-100 mg of fecal dry matter content was applied to the extraction procedure. Then, 1000 µl of 0.05 M PBS buffer in D_2_O was added. The sample was vortexed and sonicated until complete homogenization and the mixture (dispersion) was centrifuged (20,000 ***g***, 25 min at 4°C). The solution was separated into an upper clear phase and a lower turbid phase (with insoluble compounds, as lipids, proteins and cellular debris), and 600 µl of the clear upper phase was placed into a 5-mm outside diameter nuclear magnetic resonance (NMR) tube [electronic reference to access *in vivo* concentrations (ERETIC) signal, 1,0446 mM]. The ^1^H NMR spectra were recorded at 300 K on an Avance III 500 spectrometer (Bruker, Ettlingen, Germany), operating at a proton frequency of 500.20 MHz, using a 5-mm broadband observe (BBO) gradient probe. The frequency domain spectra were manually phased and baseline corrected using TopSpin software (version 3.2, Bruker). The spectral data were normalized to the total spectral area prior to data analysis. The metabolite resonances were assigned on the basis of comparison with the literature and existing databases, such as the Human Metabolome Database (HMDB, http://www.hmdb.ca/), and metabolomics toolbox (Chenomx NMRSuit 7.6, Chenomx, Edmonton, Canada). Metabolomics data analysis was performed with Metaboanalyst 4.0 platform (Chong et al., 2018). After data pre-treatment and standardization, OPLS-DA or PLS-DA models were established.

### RT-qPCR

DNA from fecal samples was amplified in a StepOnePlus Real-Time PCR system (Applied Biosystems, Weiterstadt, Germany) using SYBER Green I Master (Roche Diagnostics GmbH, Mannheim, Germany), to determine total bacterial concentration using the primers listed in Table S3. Each sample was run in triplicate and quantitative PCR standards were created by amplifying the target 16S rRNA genes from appropriate positive control strains. Standard curves were then generated to calculate the relative concentration of each sample.

To determine relative gene expression in liver and gut samples, RNA was isolated using Trizol reagent (Life Technologies, Madrid, Spain) and quantiﬁed using a NanoDrop1000 spectrophotometer (Thermo Fisher Scientiﬁc, Wilmington, DE, USA). Residual genomic DNA was removed by incubating RNA with RQ1 RNase-free DNase (Promega, Madison, WI, USA). First-strand cDNA was amplified using a High-Capacity cDNA Archive Kit (Applied Biosystems) in an Applied Biosystems 7500 Real-Time PCR system using SYBER Green I Master (Roche Diagnostics GmbH) and the primers listed in Table S3. The 2^-ΔΔCt^ method was applied to determine relative changes in gene expression levels. Glyceraldehyde-3-phosphate dehydrogenase (*Gapdh*) was used to normalize the cycle number at which the transcripts were detectable (Ct), referred to as ΔCt (Crespo et al., 2010).

### NF-κB (p65) transactivation assays

Quantitation of the expression of active NF-κB (p65) was assayed using the Thermo Scientific Transcription Factor Kit for NF-κB (p65) (Thermo Fisher Scientific, Waltham, MA, USA), as directed by the manufacturer. Briefly, liver or small intestine tissue samples were homogenized in the lysis buffer provided with the kit. Next, 10 μl of homogenized sample was further incubated with the NF-κB-binding biotinylated consensus sequence DNA (p65) attached to the streptavidin-coated 96-well plates. Only the active NF-κB p65 binds to the consensus sequence and is subsequently detected with anti-NF-κB p65 primary antibody and horseradish peroxidase (HRP)-conjugated secondary antibody and a chemiluminescent substrate (all provided in the kit). Chemiluminescence was detected in an EnVision Multilabel Plate Reader Model 2102 (Perkin Elmer, Waltham, MA, USA) and reported as relative chemiluminescence units.

### Statistical analysis

Data are expressed as the mean±s.e.m. Significant differences were evaluated by one-way analysis of variance (ANOVA) and Newman–Keul's test. *P*<0.05 was considered to be significant. Gut microbiota analysis was determined by non-parametric Kruskal–Wallis test followed by Mann–Whitney *U*-test; a false discovery rate (FDR)-adjusted *P*<0.05, was considered for significance. All statistical analyses were performed using SPSS 22.0 software (Chicago, IL, USA).

Spearman's correlation was used to examine correlations between gut microbiota composition and obesity, metabolic syndrome and NAFLD markers or fecal metabolites using the Microbiome R package (http://microbiome.github.com/microbiome). Correlations with FDR-adjusted *P*<0.05 were selected to construct the network map.

## Supplementary Material

Supplementary information
