# Supplementary material for: Beneficial effects of exercise on gut microbiota functionality and barrier integrity, and gut-liver crosstalk in an in vivo model of early obesity and non-alcoholic fatty liver disease
Source: Dis Model Mech. 2019 Apr 30;12(5):dmm039206. doi: 10.1242/dmm.039206 (PMC6550047; doi:10.1242/dmm.039206)
Supplement: Supplementary information [file dmm-12-039206-s1.pdf]

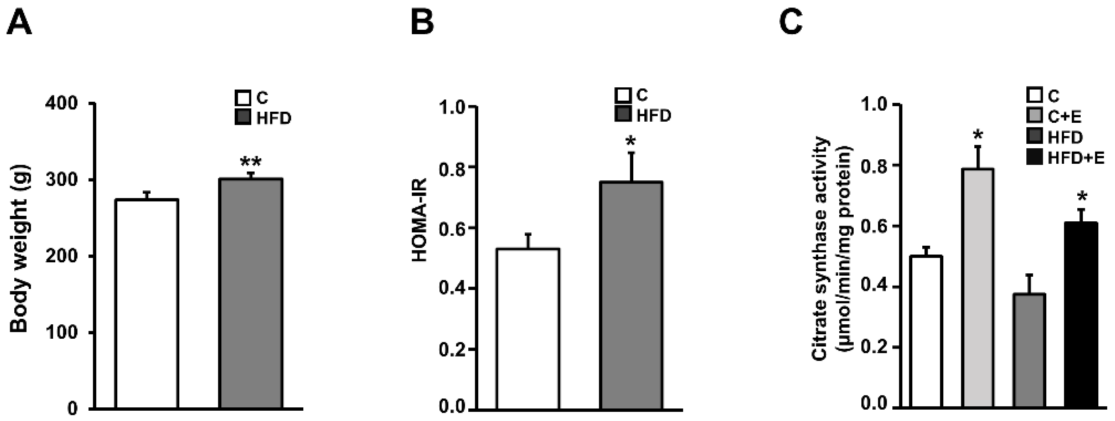

Figure S1. HFD-induced obesity and metabolic syndrome onset and exercise effectiveness in our *in vivo* model. (A) Body weight and (B) Insulin resistance (HOMA-IR) of pubertal male Wistar rats fed either with control or high-fat diet (HFD) during 6 weeks prior exercise training. (C) Citrate synthase activity measured in vastus lateralis muscle from trained and sedentary rats in the 11th week, after 5 weeks of exercise performance. Data are presented as mean ± SEM of 12 rats per group. *p< 0.05, **p< 0.01 *vs* Control; #p<0.05 *vs* HFD.

Disease Models & Mechanisms • Supplementary information

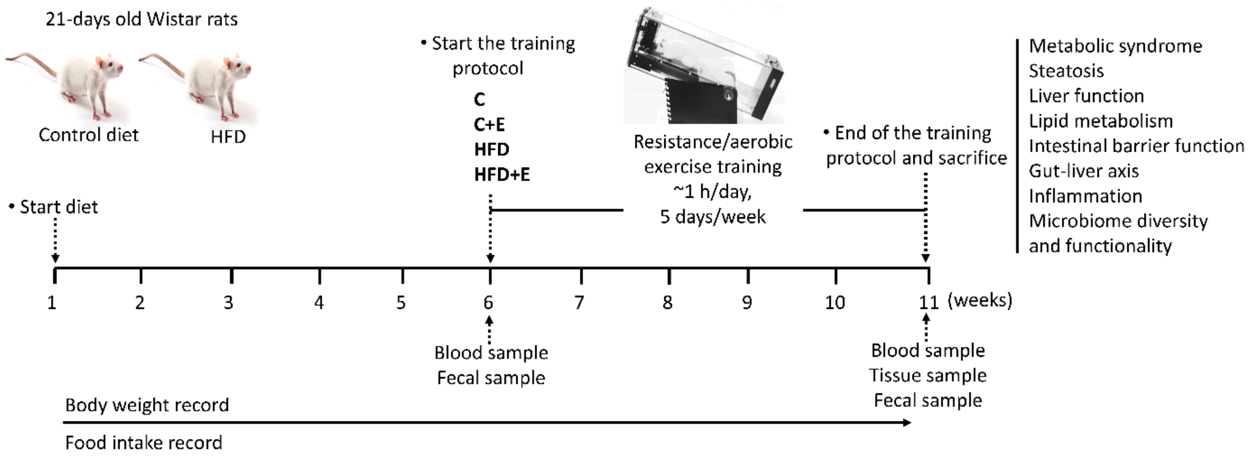

Figure S2. Experimental design of the *in vivo* model of early obesity and NAFLD. 21-days old male Wistar rats were fed with control or high fat diet (HFD) for 6 weeks followed by 5 weeks of an exercise training protocol. Body weight gain and food intake were weekly monitored. Blood and fecal samples were taken before and after the training protocol. At the end of the study rats were sacrificed to evaluate the effect of exercise in obesity/NAFLD related parameters.

Disease Models & Mechanisms • Supplementary information

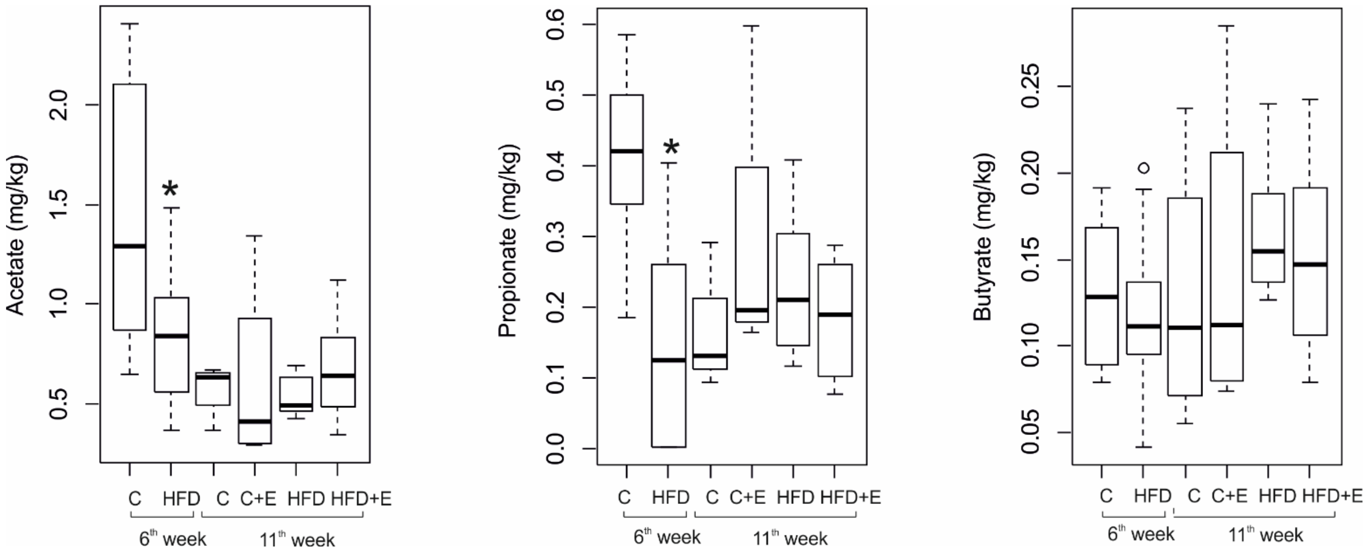

Figure S3. Fecal concentration of short chain fatty acids in rats. Box plots represent the differences related to acetate, propionate and butyrate concentrations (mg/kg) between control and HFD-fed rats with and without exercise training. Statistical analysis was performed using Kruskal-Wallis followed by Mann-Whitney U test (p<0.05). ^{a}p<0.05 *vs* Control (6th week), *p<0.05 *vs* Control.

Disease Models & Mechanisms • Supplementary information

Table S1. Animal diet composition

	D12450J		D12492	
	g (%)	kcal(%)	g(%)	kcal(%)
Protein	19	20	26	20
Carbohydrate	67	70	26	20
Fat	4	**10**	35	**60**
Total		100		100
kcal/g	3.8		5.2	
Ingredient	**g**	**kcal**	**g**	**kcal**
Casein	200	800	200	800
L-Cystine	3	12	3	12
Corn Starch	506.2	2025	0	0
Maltodextrin 10	125	500	125	500
Sucrose	68.8	275	68.8	275
Cellulose, BW200	50	0	50	0
Soybean Oil	25	225	25	225
Lard	20	180	245	2205
Mineral Mix S10026	10	0	10	0
DiCalcium Phosphate	13	0	13	0
Calcium Carbonate	5.5	0	5.5	0
Potassium Citrate, 1 H_2O	16.5	0	16.5	0
Vitamin Mix V10001	10	40	10	40
Choline Bitartrate	2	0	2	0
Yellow Dye	0.04	0	0	0
Blue Dye	0.01	0	0.05	0
Total	**1055.05**	**4057**	**773.85**	**4057**

Disease Models & Mechanisms • Supplementary information

Table S2. Details of the combined aerobic and resistance exercise training protocol performed by the training groups.

		Week 1	Week 2	Week 3	Week 4	Week 5
**Warm-up**	Inclination	0%	0%	0%	0%	0%
10 min run	Speed	15 cm/s	15 cm/s	15 cm/s	15 cm/s	15 cm/s
**Resistance training**						
Eight repeats (2-min bouts) +1-min rest	Inclination	10%	10%	15%	15%	20%
	Speed	20 cm/s	25 cm/s	20 cm/s	25 cm/s	20 cm/s
Rest 5 min						
**Aerobic training**	Inclination	10%	10%	15%	15%	20%
30 min continuous running	Speed	20 cm/s	25 cm/s	20 cm/s	25 cm/s	20 cm/s

Disease Models & Mechanisms • Supplementary information

Table S3. Primers used for RT-qPCR analysis.

	Sense primer sequence (5´-3´)	Antisense primer sequence (5´-3´)
**Rat primers**		
**CYP2E1**	ACCTTCAGTCACTGGACATCAA	AGGATCAGGAGCCCATATCTC
**LXRα**	GCACGCTACATTTGCCATAG	GAGCCTGCTCCTCTTCTTGA
**SREBP-1c**	CAGAGGGACTACAGGCTGAGAAAG	CACGTAGATCTCTGCCAGTGTTG
**FAS**	TCGGCGAGTCTATGCCACTATT	CCGAGTAATGCCGTTCAGTTC
**FAT/CD36**	GATGTGGAACCCATAACTGGA	CTTTCTCATCGCCAATGGTC
**C/EBPα**	GCCAAGAAGTCGGTGGACAAGAAC	CGGTCATTGTCACTGGTCAACTCC
**TLR-4**	TTCCTTTCCTGCCTGAGACC	CATGCCATGCCTTGTCTTCA
**TNF-α**	AAATGGGCTCCCTCTCATCAGTTC	TCTGCTTGGTGGTTTGCTACGAC
**IL-6**	TCTCTCCGCAAGAGACTTCCA	ATACTGGTCTGTTGTGGGTGG
**CLDN-1**	AGGCAACCAGACCTTGAT	CATGCACTTCATGCCAATGGTGGA
**OCLN**	TTACGGCTATGGAGGGTAC	TGACGCTGGTAACAAAGAT
**SHP**	GGCACTATCCTCTTCAACCCA	TCCAGGACTTCACACAATGCC
**FXR**	CCACGACCAAGCTATGCAG	TCTCTGTTTGCTGTATGAGTCCA
**NTCP**	AAAATCAAGCCTCCAAAGGAC	TTGTGGGTACCTTTTTCCAGA
**BSEP**	CGGTGGCTGAGAGATCAAAT	TGCGATAGTGGTGGAGAACA
**GAPDH**	GGCACAGTCAAGGCTGAGAATG	ATGGTGGTGAAGACGCCAGTA
**Bacterial primers**		
**16S rRNA 926F-1062R (total bacteria)**	AAACTCAAAKGAATTGACGG	CTCACRRCACGAGCTGAC
**16S rRNA V3-V4**	TCGTCGGCAGCGTCAGATGTGTATAA GAGACAGCCTACGGGNGGCWGCAG	GTCTCGTGGGCTCGGAGATGTGTA TAAGAGACAGGACTACHVGGGTA TCTAATCC

Abbreviations: CYP2E1, Cytochrome P450 2E1; LXRα, Liver X receptor alpha; SREBP-1c, Sterol regulatory element-binding protein 1c; FAS, Fatty acid synthase; FAT/CD36, Fatty acid translocase CD36; C/EBPα, CCAAT/enhancer-binding protein alpha; TLR-4, Toll-like receptor 4; TNF-α, Tumor necrosis factor alpha; IL-6, interleukin 6; CLDN-1, Claudin 1; OCLN, Occludin; SHP, Small heterodimer partner; FXR, Farnesoid X receptor; NTCP, Na^+/taurocholate cotransporting polypeptide; BSEP, Bile salt export pump; GADPH, Glyceraldehyde-3-phosphate dehydrogenase.

Disease Models & Mechanisms • Supplementary information
